# Activation of ATP‐sensitive potassium channels facilitates the function of human endothelial colony‐forming cells *via* Ca^2+^/Akt/eNOS pathway

**DOI:** 10.1111/jcmm.13006

**Published:** 2016-10-06

**Authors:** Yan Wu, Meng‐Yu He, Jian‐Kui Ye, Shu‐Ying Ma, Wen Huang, Yong‐Yue Wei, Hui Kong, Hong Wang, Xiao‐Ning Zeng, Wei‐Ping Xie

**Affiliations:** ^1^ Department of Respiratory MedicineWuXi People's Hospital Affiliated to Nanjing Medical UniversityWuxiJiangsuChina; ^2^ Department of Respiratory and Critical Care MedicineThe First Affiliated Hospital of Nanjing Medical UniversityNanjingJiangsuChina; ^3^ Department of BiostatisticsSchool of Public HealthNanjing Medical UniversityNanjingJiangsuChina

**Keywords:** K_ATP_, ECFCs, iptakalim, angiogenesis, Ca^2+^ signalling

## Abstract

Accumulating data, including those from our laboratory, have shown that the opening of ATP‐sensitive potassium channels (K_ATP_) plays a protective role in pulmonary vascular diseases (PVD). As maintainers of the endothelial framework, endothelial colony‐forming cells (ECFCs) are considered excellent candidates for vascular regeneration in cases of PVD. Although K_ATP_ openers (KCOs) have been demonstrated to have beneficial effects on endothelial cells, the impact of K_ATP_ on ECFC function remains unclear. Herein, this study investigated whether there is a distribution of K_ATP_ in ECFCs and what role K_ATP_ play in ECFC modulation. By human ECFCs isolated from adult peripheral blood, K_ATP_ were confirmed for the first time to express in ECFCs, comprised subunits of Kir (Kir6.1, Kir6.2) and SUR2b. KCOs such as the classical agent nicorandil (Nico) and the novel agent iptakalim (Ipt) notably improved the function of ECFCs, promoting cell proliferation, migration and angiogenesis, which were abolished by a non‐selective K_ATP_ blocker glibenclamide (Gli). To determine the underlying mechanisms, we investigated the impacts of KCOs on CaMKII, Akt and endothelial nitric oxide synthase pathways. Enhanced levels were detected by western blotting, which were abrogated by Gli. This suggested an involvement of Ca^2+^ signalling in the regulation of ECFCs by K_ATP_. Our findings demonstrated for the first time that there is a distribution of K_ATP_ in ECFCs and K_ATP_ play a vital role in ECFC function. The present work highlighted a novel profile of K_ATP_ as a potential target for ECFC modulation, which may hold the key to the treatment of PVD.

## Introduction

ATP‐sensitive potassium channels (K_ATP_), widely expressed in tissues and cells, participate in numerous cellular processes. Mounting evidence has confirmed that K_ATP_ protect myocardium against ischaemia, regulate vascular smooth muscle contraction and are involved in the secretion of insulin from pancreatic β cells 1. Recently, prevailing attention has been paid to the regulatory role of K_ATP_ in endothelial cells (ECs). As the major components of the pulmonary vasculature, ECs have been widely reported to be impaired during the progression of pulmonary vascular diseases (PVD). Several studies have revealed that K_ATP_ play a critical role in pulmonary ECs to shear stress and arterial vasomotion 2, 3. In mice, knockout of inward rectifier potassium channel 6.1 (Kir6.1), one of the pore‐forming subunits of K_ATP_, in the endothelium impaired coronary artery function and elevated blood pressure 4. In human umbilical vein endothelial cells, down‐regulation of Kir6.1 blunted cell migration and decreased neovascularization 5. Herein, K_ATP_ have been considered as a crucial controller of ECs.

Endothelial progenitor cells (EPCs), first isolated in 1997 6, are derived from bone marrow and can be found in peripheral circulation. Regarded as precursors of ECs, EPCs have been investigated extremely on its contribution to vasculogenesis and vascular homeostasis. Until now, several studies have demonstrated that transplantation of EPCs ameliorated and reversed monocrotaline (MCT)‐induced pulmonary arterial hypertension (PAH) 7, 8. Endothelial progenitor cell‐based cell therapy suppressed the development of atherosclerosis with a down‐regulation of pro‐inflammatory protein 9. Although accumulating data have indicated that EPCs may provide a circulating pool of immature ECs for endothelium regeneration, little is known about the role of K_ATP_ in the regulation of EPCs.

Endothelial colony‐forming cells (ECFCs), one type of EPC, acquire a completely endothelial phenotype and are thought to be ‘true’ EPCs 10. Endothelial colony‐forming cells display high proliferative potentials and angiogenic ability both *in vitro* and *in vivo* 11, 12. Recently, ECFCs have been used as a therapeutic tool for vascular regeneration to ultimately maintain the integrity of vessels 13, 14, 15. Many efforts are made to improve ECFC function, but few have proved effective. Endothelial colony‐forming cells injected into peripheral circulation showed low percentage (10%) of cell retention and survival within the injury site 16, 17. Initial attempts with autologous ECFC also failed to achieve prospective outcomes for the deficiency in cell function. Therefore, rescue of ECFC function has been considered as a promising strategy for PVD therapies.

Although the contribution of K_ATP_ on ECs has been confirmed before, it remains unclear whether K_ATP_ is expressed in ECFCs, and what the role K_ATP_ play in ECFC function. This study has determined for the first time that K_ATP_ are expressed in human ECFCs and is composed of Kir6.1/6.2 and sulfonylurea receptor 2b (SUR2b). Furthermore, we also revealed that K_ATP_ openers (KCOs) could promote ECFC proliferation, migration and angiogenesis by Ca^2+^/Akt/endothelial nitric oxide synthase (eNOS) signalling, which will improve our understanding on the respiratory biology of K_ATP_ and open new perspectives for ECFC management.

## Materials and methods

### Chemicals

Iptakalim (Ipt) was a gift from the Institute of Pharmacology and Toxicology, Academy of Military Medical Sciences (Beijing, China). Nicorandil (Nico) and glibenclamide (Gli) were purchased from Sigma‐Aldrich (St. Louis, MO, USA). Nico and Gli were diluted with dimethyl sulfoxide (DMSO), and Ipt was diluted with double‐distilled H_2_O. The concentration of DMSO was controlled below 0.1% (vol/vol), and did not affect the biological viability of the culture cells.

### Cell culture

Human ECFCs were cultured using a protocol approved by the First Affiliated Hospital of Nanjing Medical University. Human peripheral blood‐derived ECFCs were isolated and cultured as described previously 18, 19. Briefly, human peripheral blood (20 ml) obtained from healthy adult donors with informed consent was fractionated *via* centrifugation in a Ficoll‐Paque (Sigma‐Aldrich) gradient. Isolated mononuclear cells were re‐suspended in endothelial basal medium‐2 (EBM‐2) (Lonza, Walkersville, MD, USA), consisting of 10% foetal bovine serum (FBS), human epidermal growth factor (hEGF), human VEGF1, human fibroblast growth factor (hFGF), insulin‐like growth factor 1 (IGF‐1), ascorbic acid and heparin. Cells were diluted to a final concentration of 7.5 × 10^5^ cells per ml and seeded into 2% fibronectin‐coated (Sigma‐Aldrich) six‐well plates with approximately 2 × 10^6^ cells per well. Medium was replaced after the first 24 hrs. Under daily observation, colonies of ECFCs were obtained after 14–28 days on average. Cells were used in their fourth to sixth passages.

Human pulmonary artery endothelial cells (HPAECs) were purchased from Lonza and cultured in EBM‐2 (Lonza) supplemented with 5% FBS, hEGF, human VEGF, hFGF, IGF‐1, ascorbic acid and heparin. Both ECFCs and HPAECs were cultured in an incubator at 37°C in 5% CO_2_ saturated with H_2_O.

### Phenotype characterization

The cultured cells were defined by fluorescence‐activated cell sorting (FACS) analysis. Cells were incubated with the following fluorescent antibodies: VEGF receptor‐2 (VEGFR‐2/CD309) (Miltenyi Biotec, Bergisch Gladbach, Germany), CD34 (Miltenyi Biotec), CD133 (Miltenyi Biotec), CD45 (Miltenyi Biotec), CD14 (Miltenyi Biotec) and CD115 (BioLegend Inc, San Diego, CA, USA). Fluorescent isotype‐matched antibodies were used as negative controls. Cells were washed, fixed in paraformaldehyde and analysed on a FACS Canto II Instrument (Becton‐Dickinson, Heidelberg, Germany).

Adherent cells were tested for uptake of both DiI‐labelled acetylated low‐density lipoprotein (DiI‐ac‐LDL) (Thermo Fisher Scientific, Waltham, MA, USA) and FITC‐labelled Ulex europaeus agglutinin‐1 (FITC‐UEA‐I) (Sigma‐Aldrich). Immunofluorescence staining was performed for further molecular identification. Fixed with 4% paraformaldehyde for 30 min., cells were washed with PBS and stained with the primary antibodies CD31 (Santa Cruz, Santa Cruz, CA, USA) and von Willebrand factor (vWF; Santa Cruz) at 4°C overnight. Then, the cells were incubated with Alexa Fluor 488 donkey anti‐goat IgG (Thermo Fisher Scientific) and Alexa Flour 555 donkey anti‐rabbit IgG (Thermo Fisher Scientific). The nuclei were counterstained with 4′,6‐diamidino‐2‐phenylindole (DAPI; Sigma‐Aldrich). Double fluorescence detection is shown in colour‐merged images, captured under an LSM 510 confocal microscope (Zeiss, Oberkochen, Germany).

### Reverse transcription polymerase chain reaction

Total mRNA was extracted from ECFCs and HPAECs using the Qiagen RNeasy mini kit (Qiagen, Venlo, The Netherlands), according to the manufacturer's instructions. First‐strand cDNA was synthesized using the Prime Script RT reagent kit (TakaraBio, Shiga, Japan) and amplified by Phusion DNA Polymerase (Thermo Fisher Scientific) in a 25 μl reaction mixture. Each cDNA was subjected to PCR amplification using gene‐specific primers. The primer sequences for each of the K_ATP_ subtypes and the actin housekeeping genes are listed in Table 1. PCR was conducted using a PCR system Master Cycler. Thermal cycling conditions for all PCR mixtures were as follows: initial denaturation for 30 sec. at 98°C; 40 cycles of 10 sec. at 98°C (denaturation), 30 sec. at 58°C (annealing) and 30 sec. at 72°C (extension); and a final extension for 10 min. at 72°C. PCR mixtures were subsequently separated and analysed on a 1.5% agarose gel. The mRNA expression of actin was utilized as an internal control. HPAECs and mouse brain were used as positive controls. Water was used as a negative control.

**Table 1:** Primers and product sizes for RT‐PCR

Target gene	Primer sequence	Size (bp)
Kir6.1	F:GGAGGATGATGACAGAGGAATGC	313
	R:GGAATGTCCAGTTGGTGAATAGG	
Kir6.2	F:TCCAAGAAAGGCAACTGCAACGT	1063
	R:AGGACAGGGAATCTGGAGAGATGCT	
SUR1	F:TGCCGCACGTCTTCCTACTCTTCA	609
	R:TGAAGGCGTTCATCCACCAGTAGGT	
SUR2a	F:TCAGGACGGATTATCTGGGAGCTT	895
	R:AAGACTAAAACAAGGCCTGCATCCA	
SUR2b	F:AGGACGGATTATCTGGGAGCTTGC	941
	R:GCCAAGAGGCTTTCTGGAGTGTCA	
Actin	F:ACACCTTCTACAATGAGCTGCGTGT	741
	R:GCTCAGGAGGAGCAATGATCTTGA	

Kir6.1: inward rectifier potassium channel 6.1; Kir6.2: inward rectifier potassium channel 6.2; SUR1: sulfonylurea receptor 1; SUR2a: sulfonylurea receptor 2a; SUR2b: sulfonylurea receptor 2b; F: forward; R: reverse.

### Western blotting analysis

Endothelial colony‐forming cells were treated with Ipt (10^−4^ M), Nico (10^−4^ M) or Gli (10^−5^ M) for 30 min. To extract total protein, ECFCs and HPAECs were lysed with radioactive immunoprecipitation assay buffer (150 mM NaCl, 50 mM Tris–HCl, 5 mM ethylenediaminetetraacetic acid, 0.1% SDS, 1% TritonX‐100, 1 protease inhibitor cocktail, 1 mM phenylmethanesulfonyl fluoride (PMSF)). Lysates were clarified *via* centrifugation at 13,800 g for 15 min. at 4°C. Protein concentrations were determined using a BCA Protein Assay kit (Beyotime, Nantong, China). The samples were separated by SDS‐PAGE and transferred onto poly‐vinylidenedifluoride membranes (Millipore, Thermo Scientific, Sunnyvale, CA, USA). The membranes were blocked with Tris‐buffered saline containing 0.05% Tween 20 and 5% bovine serum albumin (BSA) for 1 hr. After blocking, the transferred membranes were incubated with primary antibodies against Kir6.1 (Alomone Labs, Jerusalem, Israel), Kir6.2 (Abcam, Cambridge, UK), SUR1 (Abcam), SUR2a (Santa Cruz), SUR2b (Santa Cruz), calcium/calmodulin‐dependent protein kinase II (CaMKII) (Santa Cruz), phospho‐CaMKII (P‐CaMKII) (Santa Cruz), Akt (Cell Signaling Technology, Danvers, MA, USA), phospho‐Akt (P‐Akt) (Cell Signaling Technology), eNOS (Santa Cruz), phospho‐eNOS (P‐eNOS) (Ser1177; Santa Cruz) and GAPDH (Santa Cruz). Then, the blot was incubated with the appropriate horseradish‐peroxidase (HRP)‐conjugated rabbit IgG (Santa Cruz) or HRP‐conjugated goat IgG as a secondary antibody (Santa Cruz). Total mouse brain proteins were used as positive controls. The signals were visualized using an enhanced chemiluminescence reagent kit (Thermo Fisher Scientific) and analysed with Bio‐Rad Gel Doc/Chemi Doc Imaging System and Quantity One software (Hercules, Millipore, Billerica, MA, USA).

### Confocal microscopy

In brief, after fixation and blocking, ECFCs were permeabilized with 0.5% Triton X‐100 (Sigma‐Aldrich) in PBS, blocked with 5% BSA for 1 hr at room temperature and incubated with antibodies against Kir6.1 (Alomone Labs), Kir6.2 (Abcam), SUR2b (Santa Cruz), CD31 (Santa Cruz) and VE‐cadherin (Abcam) at 4°C overnight. After three washes with PBS, ECFCs were incubated with secondary antibodies as stated above for 1 hr at room temperature in the dark. DAPI was used for nuclei staining. Cells were visualized using an LSM 510 confocal microscope (Zeiss).

### Cell proliferation assays

The incorporation of 5‐ethynyl‐2′‐deoxyuridine (EdU) with the EdU Cell Proliferation Assay Kit (Ribobio, Guangzhou, China) was used for detecting cell proliferation. All the procedures were performed according to the manufacturer's protocol. In brief, cells were pretreated with Ipt (10^−6^, 10^−5^ and 10^−4^ M) or Nico (10^−6^, 10^−5^ and 10^−4^ M) in EBM‐2 containing 2% FBS for 24 hrs and then incubated with 50 μM EdU for 4 hrs before fixation, permeabilization and EdU staining. To investigate the interaction between KCOs and K_ATP_ blockers on human ECFCs, Gli (10^−5^ M) was added 30 min. prior to the addition of Ipt and Nico. The EdU incorporation rate was determined as the ratio of the EdU‐positive cells (red cells) to the total Hoechst 33342‐positive cells (blue cells). Five fields were randomly selected from each dish by fluorescence microscopy (DM2500; Leica, Wetzlar, Germany) and at least three dishes were counted per treatment.

Cell counting kit‐8 (CCK8) assay was performed to further confirm the effect of KCOs on the proliferation of human ECFCs. Human ECFCs were seeded into a 96‐well plate at a density of 10,000/well overnight. Then, cells were treated with Ipt (10^−6^, 10^−5^ and 10^−4^ M) or Nico (10^−6^, 10^−5^ and 10^−4^ M) with EBM‐2 containing 2% FBS for 24 hrs. Cells were pre‐incubated with Gli (10^−5^ M) for 30 min. before adding Ipt and Nico. Cell proliferation was analysed with CCK‐8 kits (Dojindo Molecular Technologies, Kumamoto, Japan) according to the manufacture's protocol. Absorbency was measured at 450 nm by a microplate reader (Thermo Scientific, Millipore, Billerica, MA, USA).

### Cell migration

We used transwell migration chamber assays to examine ECFC migration. Migration assays were performed using a 24‐well Transwell chamber (Corning, Corning, NY, USA). Cells were re‐suspended in serum‐free EBM‐2 at 1 × 10^5^ cells/ml and a 100‐μl cell suspension was added to the upper chamber. Endothelial basal medium‐2 containing 10% FBS in the presence or absence of different concentrations of Ipt or Nico (10^−6^, 10^−5^ and 10^−4^ M) were added into the lower chamber. Gli (10^−5^ M) was added 30 min. before the addition of other drugs. The chamber was incubated for 24 hrs at 37°C. The filter was carefully removed and cells attached on the upper side were wiped off. Cells migrating through the filter and appearing on the lower side were fixed with 4% paraformaldehyde for 20 min. and stained with 5% crystal violet. We randomly selected five vision fields and counted cells using a phase‐contrast microscope (Nikon, Tokyo, Japan) for statistical analysis 20.

### *In vitro* tube formation on Matrigel plates

Endothelial colony‐forming cell and HPAEC angiogenesis were evaluated by an *in vitro* tube formation assay. Ten microlitres of Matrigel (Becton‐Dickinson, San Diego, CA, USA) basement membrane matrix was added to a u‐slide angiogenesis plate (Ibidi, Martinsried, Germany). The slide was incubated at 37°C for 30 min. to allow the matrix solution to solidify. Cells were diluted to a final concentration of 1.5 × 10^5^ cells per ml in basal medium containing 2% FBS. Fifty microlitres of cell suspension was seeded onto the surface of the u‐slide well pre‐coated with 10 μl Matrigel in the presence or absence of different concentrations of Ipt (10^−6^, 10^−5^ and 10^−4^ M) or Nico (10^−6^, 10^−5^ and 10^−4^ M). Gli (10^−5^ M) was added 30 min. prior to the treatment of Ipt and Nico. After 4 hrs of incubation, we randomly chose four fields from an unbiased examiner in each plate. The length of complete tubes per unit area was calculated using Image‐Pro Plus 20.

### Statistical analysis

All data were shown as mean ± S.D. Comparisons between controls and samples treated with various drugs were analysed by one‐way anova. Least significant difference *post hoc* tests were conducted when significant effects were found. Statistical significance was defined as *P* < 0.05.

## Results

### Characterization of ECFCs derived from human peripheral blood

**Figure 1:**
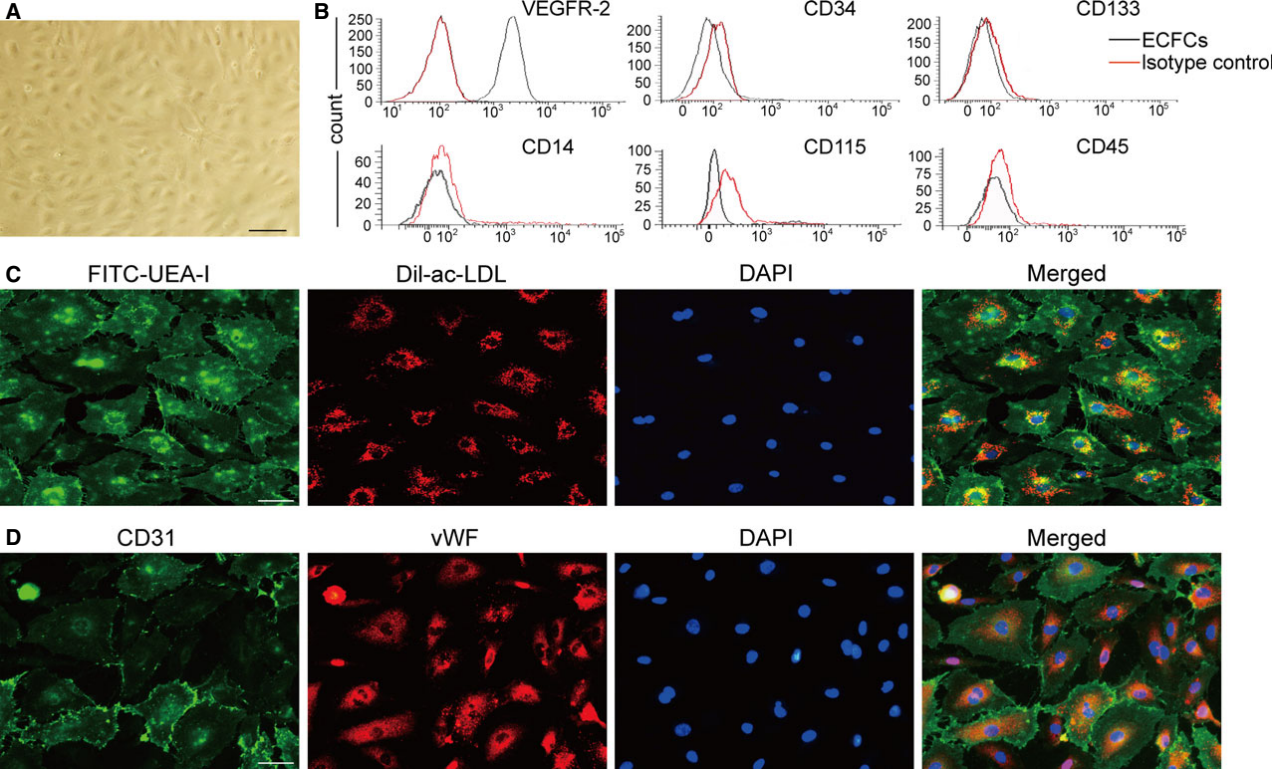
Characterization of ECFCs derived from human peripheral blood. (**A**) After 14–28 days of culture, cells presented a typical cobblestone shape as shown by a phase‐contrast inverted microscope. (**B**) FACS analysis found that most of the cells expressed VEGFR‐2, a small percentage of cells expressed CD34 and few expressed CD133, CD14, CD45 and CD115 (isotype control IgG staining shown in shaded red). (**C**) Testing of DiI‐ac‐LDL endocytose and FITC‐UEA‐1 binding showed that the cells were double positive. (**D**) Immunofluorescence staining demonstrated that the cells expressed CD31 and vWF, counterstaining with DAPI for nucleus, scale bar: 50 μm.

Endothelial colony‐forming cells were isolated and expanded *ex vivo* from adult peripheral blood. Most of the ECFC colonies appeared during 14–28 days. Initially, small colonies were observed; then, they rapidly expanded into large colonies. The cells showed characteristic endothelial ‘cobblestone’ morphology, providing easy preliminary identification under an optical microscope (Fig. 1A). Several experiments were conducted for further identification. FACS results showed that 99.5% of the isolated cells expressed the endothelial marker VEGFR‐2 (CD309); a small percentage of cultured cells expressed CD34 (3%). Nevertheless, CD133, CD15, CD45 and CD115 were almost absent in the obtained cells, which were consistent with previous reports 21 (Fig. 1B). Additionally, the cells displayed an endothelial phenotype: they took up DiI‐ac‐LDL, bound to FITC‐UEA‐I (Fig. 1C), and stained positively for the endothelium‐specific antigens CD31 and vWF (Fig. 1D).

### Determination of K_ATP_ expression in ECFCs

**Figure 2:**
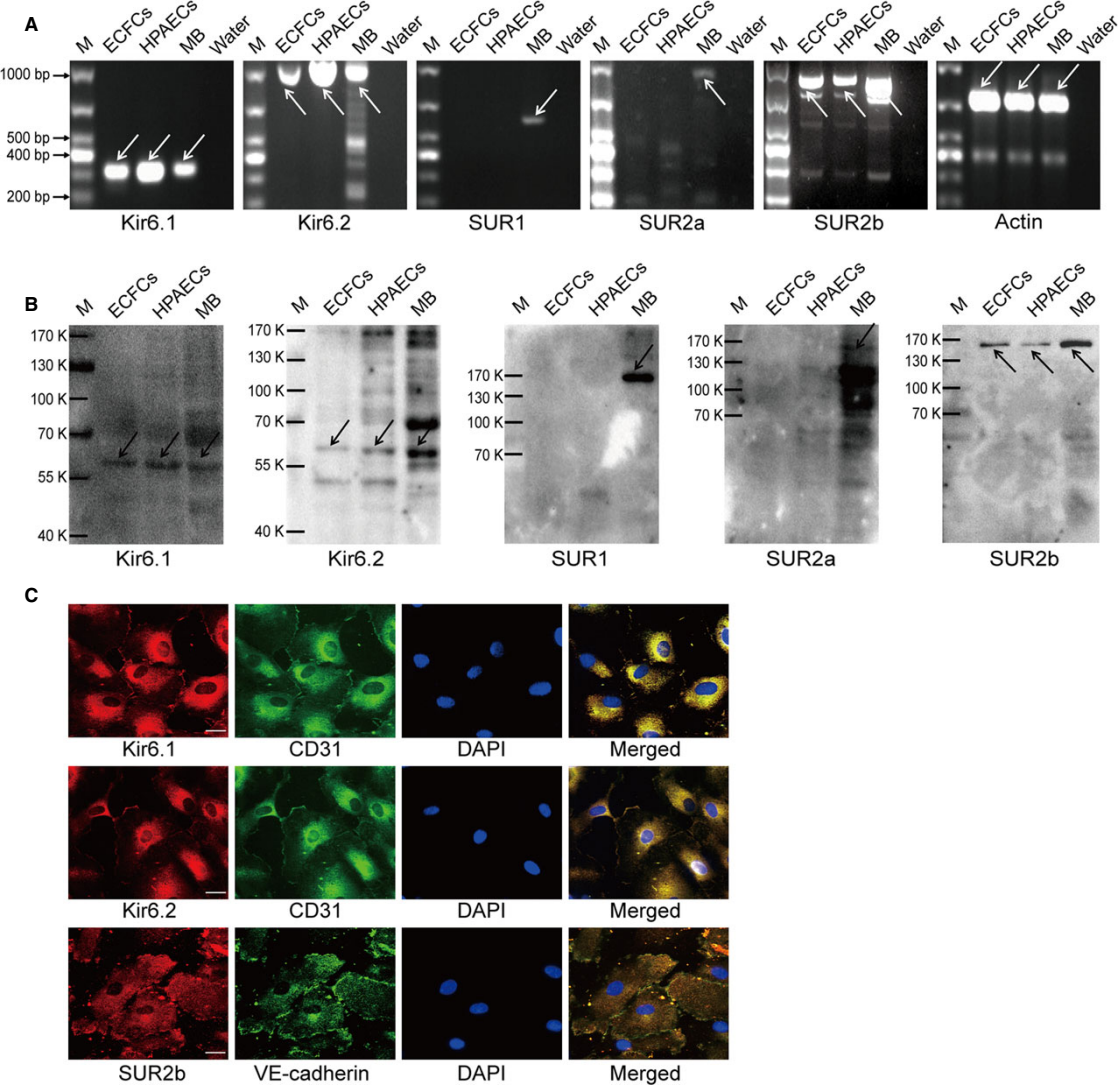
The expression of K_ATP_ subtypes in ECFCs. (**A**) RT‐PCR showed the expression of Kir6.1, Kir6.2 and SUR2b, but not SUR2a and SUR1 in mRNA level. HPAECs and mouse brain were used as positive controls, water (no template) as a negative control (*n* = 3). (**B**) Western blotting confirmed the expression of Kir6.1 (48 kD), Kir6.2 (44 kD) and SUR2b (140–150 kD), but not SUR2a (140–150 kD) and SUR1 (175 kD), using HPAECs and mouse brain as positive controls (*n* = 3). (**C**) Confocal images showed the subcellular localization of K_ATP_ subunits in ECFCs co‐stained with a endothelial specific marker (CD31 or VE‐cadherin), revealing the extensive distribution of Kir6.1, Kir6.2 and SUR2b. DAPI staining for nuclear labelling (*n* = 3), scale bar: 20 μm. M: marker, MB: mouse brain.

To determine the expression of K_ATP_ in ECFCs, we performed reverse transcription polymerase chain reaction (RT‐PCR) and western blotting for the associated mRNA and protein, using HPAECs and mouse brain as positive controls. The mRNA of Kir6.1, 6.2 and SUR2b was detected in human ECFCs, but not SUR1 and SUR2a (Fig. 2A). These subunits were further confirmed in protein level by western blotting. As shown in Figure 2B, the expression of Kir6.1, Kir6.2 and SUR2b was determined.

### Subcellular localization of K_ATP_ subunits in ECFCs

Confocal images were gained to identify the distribution of K_ATP_ in ECFCs co‐stained with an endothelium‐specific marker and showed that Kir6.1 and Kir6.2 were expressed not only in the cell membrane but also in the cytoplasm (Fig. 2C). SUR2b was also detected in the cytoplasm and intracellular patterns of punctuated and filamentous staining were observed (Fig. 2C).

### KCOs promoted ECFC proliferation, migration and angiogenesis

**Figure 3:**
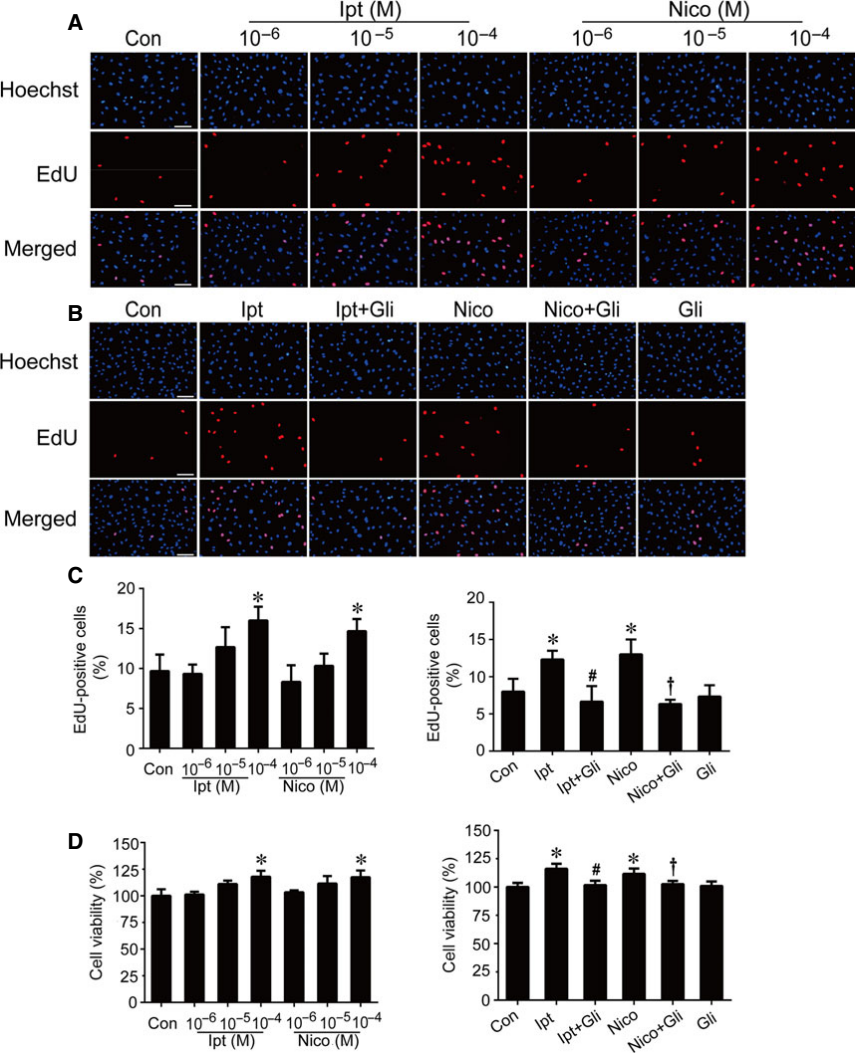
Effects of Ipt and Nico on ECFC proliferation. The number of EdU‐positive cells was elevated by treating with Ipt and Nico (**A** and **C**) The results of CCK8 showed that Ipt and Nico enhanced the viability of ECFCs (**D**). The increasing effects of Ipt and Nico were abolished by Gli (**B**–**D**). Data were shown as mean ± S.D. EdU incorporation analysis and CCK8 assay were performed three times. Statistically significant differences: *P < 0.05 *versus* control group; #*P* < 0.05 Ipt group *versus* Ipt+Gli group; †*P* < 0.05 Nico group *versus* Nico+Gli group, scale bar: 100 μm.

**Figure 4:**
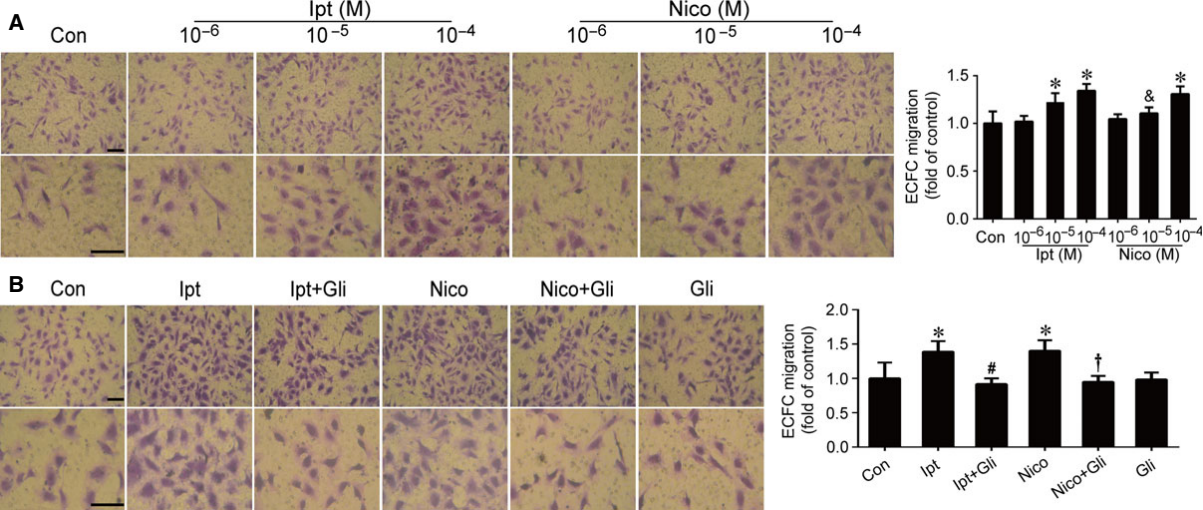
Effects of Ipt and Nico on ECFC migration. Ipt and Nico increased ECFC migration and Ipt was more effective than Nico at the concentration of 10^−5^ M (**A**). The increase originated from KCOs could be abolished by Gli (**B**). Data were shown as mean ± S.D. of four independent experiments. Statistically significant differences: *P < 0.05 *versus* control group; &*P* < 0.05 Ipt 10^−5^ M *versus* Nico 10^−5^ M; #*P* < 0.05 Ipt group *versus* Ipt+Gli group; †*P* < 0.05 Nico group *versus* Nico+Gli group, scale bar: 50 μm.

**Figure 5:**
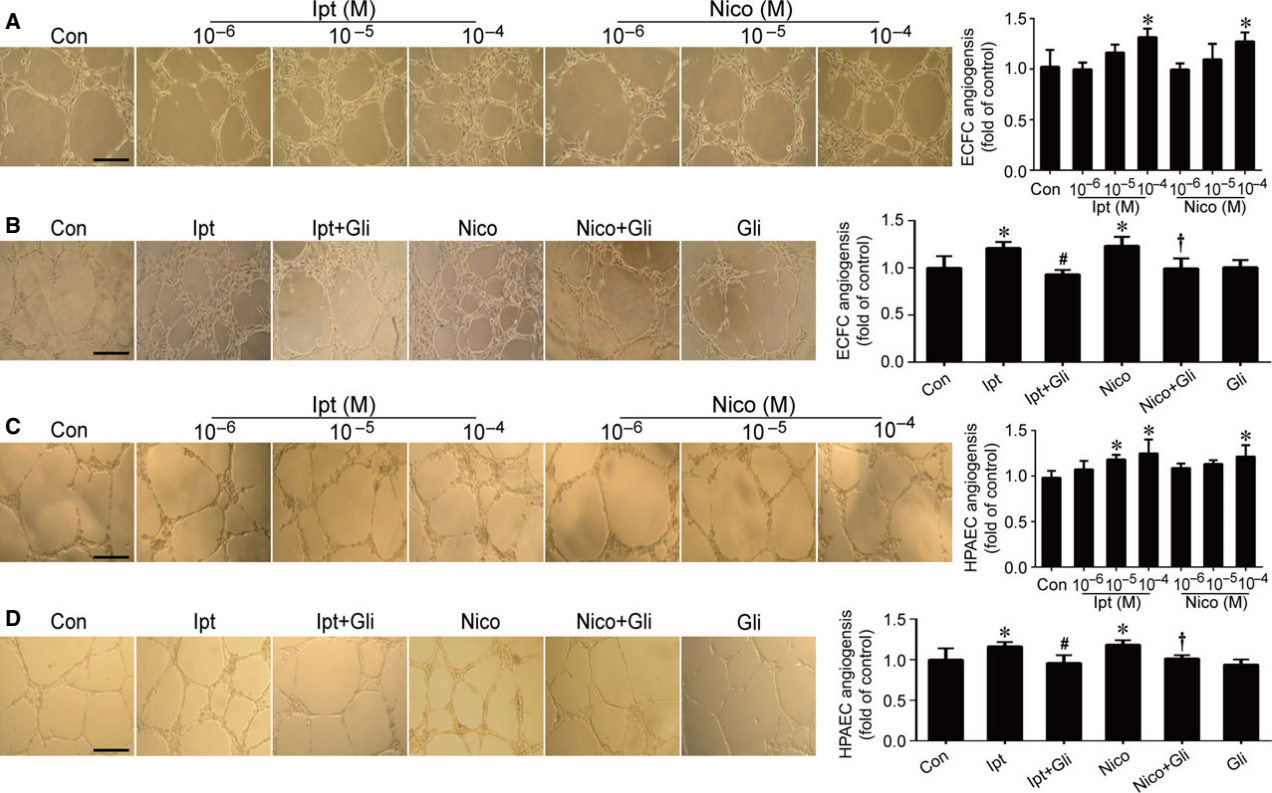
Effects of Ipt and Nico on ECFC and HPAEC angiogenesis. The results showed the promotion of Ipt and Nico in ECFC (**A**) and HPAEC (**C**) angiogenesis. Gli abolished Ipt‐ and Nico‐induced ECFC (**B**) and HPAEC (**D**) angiogensis. Data were shown as mean ± S.D. of four independent experiments. Statistically significant differences: *P < 0.05 *versus* control group; #*P* < 0.05 Ipt group *versus* Ipt+Gli group; †*P* < 0.05 Nico group *versus* Nico+Gli group, scale bar: 100 μm.

As shown in Figures 3, 4, 5, activation of K_ATP_ by KCOs significantly promoted ECFC function such as cell proliferation, migration and angiogenesis. The data from EdU incorporation assay showed that the number of EdU+ cells was markedly elevated by Ipt (10^−4^ M) and Nico (10^−4^ M) (*versus* control group, *P* < 0.05) (Fig. 3A and C). The effects of KCOs on ECFC proliferation was further substantiated by CCK8 assay. The data manifested that cell viability was elevated by Ipt (10^−4^ M) and Nico (10^−4^ M) (*P* < 0.05 *versus* control group) (Fig. 3D). As a novel KCO, the treatment of Ipt in promoting ECFC proliferation presented as good as that of Nico, as there was no significant difference between Ipt and Nico at the concentration of 10^−4^ M.

Data from transwell migration assay indicated that KCOs notably improved ECFC migration with more permeation compared with control. Treatment with 10^−5^ M Ipt and 10^−4^ M Ipt could significantly facilitate the migration of ECFCs, whereas treatment with 10^−4^ M Nico could markedly promote the ability of migration (Fig. 4A). Ipt was more effective than Nico that 10^−5^ M Ipt had already indicated its work on promoting ECFC migration (Ipt 10^−5^ M *versus* Nico 10^−5^ M, *P* < 0.05); however, there was no difference between Nico and Ipt at the concentration of 10^−4^ M.

Moreover, neovascularization is considered as one of the essential functions of ECFCs. Angiogenesis is recognized as the basic step of developing new capillaries *in vivo*. Using an *in vitro* model of angiogenesis, we found that KCO treatments facilitated the capacity of ECFCs and HPAECs to form capillary‐like structures. Our findings showed that Ipt and Nico markedly promoted ECFC angiogenesis at 10^−4^ M (*versus* control group, *P* < 0.05) (Fig. 5A), whereas Ipt at 10^−5^ M and Nico at 10^−4^ M notably improved HPAEC angiogenesis (*versus* control group, *P* < 0.05) (Fig. 5C).

Furthermore, ECFCs were pretreated with Gli (10^−5^ M), a non‐selective K_ATP_ blocker 22, before the administration of KCOs. The results indicated that Gli almost completely abolished the effects of Ipt and Nico, but not affected the basal levels in ECFC proliferation, migration or angiogenesis (Figs 3B–D, 4B and 5B). Moreover, Gli reversed Ipt (10^−5^ M) and Nico (10^−4^ M) induced angiogenesis of HPAECs (Fig. 5D).

### KCOs enhanced the phosphorylation of CaMKII, Akt and eNOS in ECFCs

**Figure 6:**
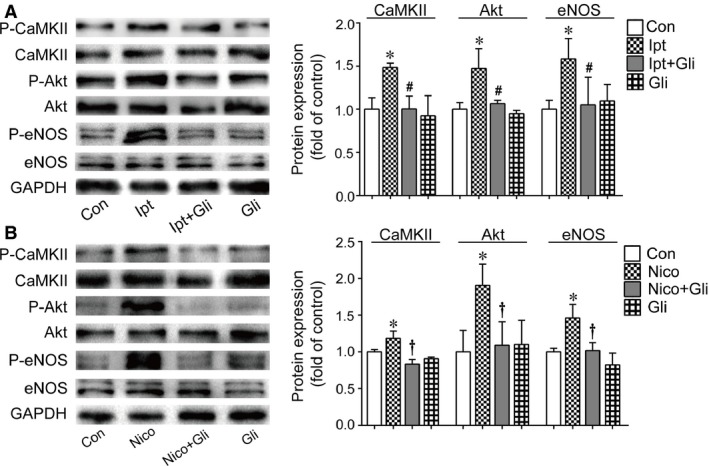
Effects of KCOs on CaMKII, Akt and eNOS. The results demonstrated the up‐regulation of phosphorylation of CaMKII, Akt and eNOS induced by Ipt (**A**) and Nico (**B**). Gli reversed the effects of Ipt and Nico on protein phosphorylation. At least three independent experiments were conducted. Statistically significant differences: *P < 0.05 *versus* control group; #*P* < 0.05 Ipt group *versus* Ipt+Gli group; †*P* < 0.05 Nico group *versus* Nico+Gli group.

Western blotting was performed to quantify the phosphorylation of intracellular signal transduction. KCO administration notably increased the phosphorylation of CaMKII, Akt and eNOS in ECFCs (Fig. 6A and B). Pretreatment with Gil significantly reversed the enhancement elicited by KCOs, while no alteration was observed with Gli alone.

## Discussion

Since first being isolated from peripheral blood 6, EPCs have gained increasing attention as a potential reparative cell therapy. Until now, the identification and characterization of EPCs have remained controversial. Based on surface markers and the culture methods used, circulating EPCs have been divided into three groups: colony‐forming unit ECs (CFU‐ECs), circulating angiogenic cells (CACs) and ECFCs 10, 23. Compared with ‘early’ EPCs, CFU‐ECs and CACs, ECFCs are considered as ‘late’ EPCs or ‘late’ outgrowth ECs. Endothelial colony‐forming cells are characterized by the absence of hematopoietic and myeloid markers, a hierarchy of proliferative potentials and the capacity for vascular formation 24. All of these features imply that ECFCs are genuine EPCs. In the current study, we isolated ECFCs from adult peripheral blood. The FACS analysis results showed the expression of EC surface antigen VEGFR‐2 but not CD133, CD14, CD45 and CD115 in ECFCs, indicating that no/minimal ‘early’ EPC contamination. However, only a small percentage of cells expressed CD34. Although some studies have shown that ECFCs from peripheral blood express CD34, there is no evidence suggesting that CD34 is necessary for ECFCs 10.

Studies have identified the expression of potassium channels in ECFCs 25, 26. However, no data are available on whether there is a K_ATP_ distribution on ECFCs and what role K_ATP_ play in the regulation of ECFCs. K_ATP_ were originally detected in the cardiovascular system in 1983 27. Serving as a molecular biosensor, K_ATP_ provide a unique connection between cellular metabolic status and membrane excitability 28. K_ATP_ are a hetero‐octameric complex consisting of a Kir6.x (Kir6.1, Kir6.2) pore and a regulatory SUR subunit (SUR1, SUR2a and SUR2b) 29. K_ATP_ can be assembled by diverse combinations of the Kir6 and SUR subunits, such as the combinations in skeletal muscle cells (SUR2a/Kir6.2), pancreatic β cells (SUR1/Kir6.2) 30 and coronary artery ECs (SUR2/Kir6.1/Kir6.2) 29. In the present study, the composition of K_ATP_ was shown to include Kir6.1, Kir6.2 and SUR2b. However, neither SUR1 nor SUR2a was detected in ECFCs. The results of confocal imaging revealed that K_ATP_ were distributed in both the cellular membrane and the cytoplasm, which may suggest a contribution of K_ATP_ in the function of subcellular organelles in ECFCs.

Studies have shown that distinct KCOs present diverse subtype selectivity. Classic KCOs include Nico and diazoxide, while Ipt is regarded as one of the novel agents. It has been shown that Nico is a hybrid agent with two pharmacologic actions, the opening of K_ATP_ and the donation of nitrogen oxide, which specifically activates K_ATP_, consisting of Kir6.1 and SUR2b 31. Because the expression of K_ATP_ in ECFCs, composed of SUR2b/Kir6.1/Kir6.2, has been confirmed by our results, Ipt and Nico were used to investigate the role of K_ATP_ in the modulation of ECFC function. As a new KCO, Ipt exhibits high selectivity for the SUR2b/Kir6.1 subtype in the previous study 32. In the current study, both Ipt and Nico significantly increased cellular proliferation, migration and angiogenesis in ECFCs, which are involved in ECFC repair and neovascularization 33. Moreover, Ipt as well as Nico presented notable superiority in promoting ECFCs proliferation and angiogenesis. However, higher efficiency of Ipt was showed for facilitating ECFC migration at the concentration of 10^−5^ M compared with Nico. Although Nico has an additional potential for nitrate properties, few effect of nitrogen oxide was observed in ECFC regulation, specifically the inhibition of KCO effects by the K_ATP_ inhibitor. Taken together, our results suggest that various KCOs exhibit diverse effects on the modulation of ECFCs.

It is well known that KCOs have protective effects on pulmonary vessels in many pathological conditions, such as hypoxia and ischaemia. *In vivo* studies have shown that KCOs are capable of reversing hypoxia‐induced pulmonary arterial remodelling and reducing right ventricular hypertrophy 34. KCOs have been shown to improve the function of smooth muscle cells (SMCs) by inducing membrane hyperpolarization and relaxing vessels 35. KCOs were also shown to have beneficial effects in ECs, including decreased hypoxia‐induced apoptosis, promotion of nitrogen oxide release and inhibition of endothelin‐1 (ET‐1) synthesis 36, 37. In our work, we demonstrated for the first time that KCOs promoted the function of ECFCs, which may uncover a new mechanism involved in the pulmonary vascular protection caused by KCOs.

Accumulating data suggest that ECFCs are a new candidate for cell‐based therapies and a new biomarker of disease progression. The number of circulating EPCs is decreased in various disorders, including chronic obstructive pulmonary disease, ischaemic chronic heart failure, coronary heart disease, rheumatoid arthritis and systemic lupus erythematosus, indicating abnormal EPC function 38, 39, 40, 41, 42. Moreover, the excessive mobilization, differentiation and maintenance of mature eosinophil progenitors induced by dysfunctional ECFCs have been shown to result in the development of asthma 43. Additionally, transplantation of EPCs failed to ameliorate the pathobiological changes in hypoxia‐induced PAH, specifically the defective migration and incorporation of EPCs into pulmonary vasculature 44. These studies have indicated that ECFCs play an integral role in numerous disorders, especially in respiratory diseases. Considering the results of the present study, improving ECFC function using KCOs could be a promising option for PVD management.

**Figure 7:**
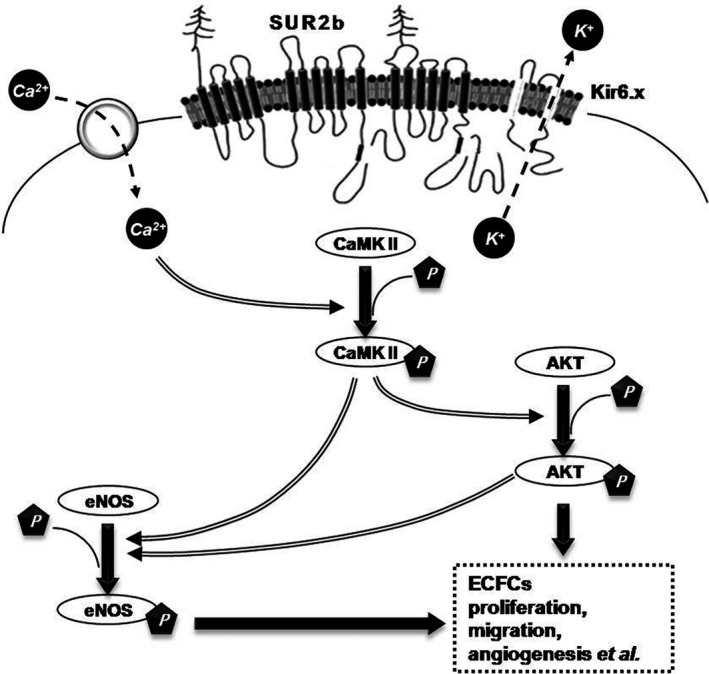
Schematic diagram of K_ATP_ regulation of ECFC proliferation, migration and angiogenesis. Opening K_ATP_ leads to the influx of Ca^2+^ in ECFCs, as a result of K^+^ efflux. The increase of Ca^2+^ up‐regulates CaMKII activation. Furthermore, P‐CaMKII leads to a high phosphorylated level of Akt and eNOS. Meanwhile, P‐Akt induces activation of eNOS in ECFCs. Ca^2+^/Akt/eNOS pathway exhibits an effect on improving ECFC proliferation, migration and angiogenesis.

Previous studies have demonstrated that the protective effect of KCOs is associated with calcium signalling 36, 45. Ca^2+^ is considered a critical node in the signalling network, and it has been implicated in a wide range of cellular processes, including cell proliferation, apoptosis, migration and gene expression 46, 47. In podocytes, excessive intracellular Ca^2+^ induced apoptosis and the effacement of foot processes 48. In ECs, Ca^2+^ influx regulated cell migration and the synthesis of vasoactive substances, such as ET‐1, nitrogen oxide and prostacyclin 49. Our previous study found that the decline of intracellular Ca^2+^ abolished platelet‐derived growth factor‐BB‐induced airway SMCs dysfunction 50. Ca^2+^ influx activates a range of downstream signalling effectors, of which CaMKII is an important molecule. The phosphorylation of CaMKII is an essential early event in Ca^2+^ signal transduction that regulates the activation of Akt and eNOS 51, 52, 53. Activation of CaMKII is required for H_2_O_2_‐induced phosphorylation of Akt in vascular smooth cells. The phosphatidylinositol 3‐kinase (PI3K)/Akt pathway is considered a regulator of cell proliferation and survival. Numerous studies have confirmed that the active PI3K/Akt pathway enhances the proliferation of numerous cells, including ECFCs 54. Moreover, Akt is one of the determinants of eNOS phosphorylation at Ser‐1177. As a Ca^2+^/CaM‐dependent enzyme, the phosphorylation of eNOS can be increased when Ca^2+^ binds to calmodulin, which is mediated by CaMKII. It has been shown that insulin up‐regulates the phosphorylation of eNOS in kidney collecting duct cells, leading to an increased production of nitrogen oxide and reduced hypertension in the insulin‐resistant state 55. The over‐expression of eNOS in EPCs that were delivered to patients with PAH could achieve notable hemodynamic improvements, implying a crucial role for eNOS in ECFCs during PVD therapy 56. These findings indicate that KCOs remarkably improve ECFC function because of the evaluated phosphorylation of CaMKII, Akt and eNOS, which could in turn be inhibited by a K_ATP_ blocker. Altogether, we suggest that Ca^2+^/Akt/eNOS signalling is involved in the regulation of K_ATP_ in ECFCs (Fig. 7).

In conclusion, this study confirmed for the first time that human ECFCs obtained from adult peripheral blood express K_ATP_, which contains the Kir6.1, Kir6.2 and SUR2b subunits. Additionally, this is the first demonstration that opening K_ATP_ with Ipt and Nico facilitates ECFC proliferation, migration and angiogenesis. Ca^2+^/Akt/eNOS signalling plays a key role in the regulation of K_ATP_ in ECFCs. Because opening K_ATP_ contributes to an improvement in ECFC function, our findings provide an effective target for ECFC regulation, thus constituting a promising therapeutic option for PVD.

## Conflict of interest

The authors confirm that there are no conflicts of interest.
